# Distinguishing Strain Field Visualization Stability from Condition-Level Incremental Strain Accuracy in High-Temperature 2D Digital Image Correlation

**DOI:** 10.3390/s26196333

**Published:** 2026-10-07

**Authors:** Yan Wang, Xiaoshan Ma, Xuan Zhang, Xiaofang Yang, Yu Pei, Huilong Zheng

**Affiliations:** 1 Institute of Engineering Thermophysics, Chinese Academy of Sciences, Beijing 100190, China; wangyan@iet.cn (Y.W.); maxiaoshan@iet.cn (X.M.); zhangxuan@iet.cn (X.Z.); yangxiaofang@iet.cn (X.Y.); 2 School of Space Exploration, University of Chinese Academy of Sciences, Beijing 100049, China; 3 National Key Laboratory of Science and Technology on Advanced Light-Duty Gas Turbine, Beijing 100190, China

**Keywords:** digital image correlation, high-temperature strain measurement, strain field visualization stability, magnification-induced apparent strain, physics-guided calibration, synthetic speckle validation

## Abstract

High-temperature two-dimensional digital image correlation (2D-DIC) can produce spatially unstable strain fields and biased condition-averaged incremental strains, but these error signatures need not respond to the same processing. A smoother strain field is therefore not, by itself, evidence of improved scalar accuracy. We evaluated strain field visualization stability separately from extensometer-referenced, condition-level incremental strain accuracy. An MA956 specimen was tested at 600, 700 and 800 °C under 12 loading conditions spanning 500–2000 µε, with four manually triggered snapshots acquired per condition. The field path used affine registration, pixelwise four-snapshot median fusion and strain radius sensitivity analysis. The scalar path used only the original unfused images and compared four prespecified models under leave-one-temperature-out internal assessment. Increasing the strain radius from 10 to 15 grid points reduced the spatial standard deviation and *P*_95_-to-median span by 22.7% and 16.6%, respectively, with little change in scalar RMSE. Controlled simulations with known ground truth showed that local non-affine disturbances primarily increased field dispersion, whereas isotropic scale changes induced approximately common-mode axial and transverse strain drift. Across 20 mechanical strain–magnification conditions, a common-mode-suppressed axial–transverse feature reduced RMSE from 439.7 to 49.8 µε. Guided by this mechanistic evidence, combining the feature with temperature terms yielded the lowest pooled out-of-fold RMSE of 76.27 µε in the experimental data, 69.7% below the raw axial strain baseline. Within the conditions examined, these results demonstrate that improved strain field visualization is insufficient evidence of scalar accuracy and support separate validation of field stability and condition-level incremental strain error.

## 1. Introduction

Reliable deformation and strain measurements under thermomechanical loading are needed to assess materials and components used in aero-engine hot sections and other high-temperature systems. Contact methods, such as strain gauges and extensometers, can provide accurate local measurements but are constrained by temperature tolerance, installation requirements, finite gauge length and restricted access. Digital image correlation (DIC) estimates displacement and strain fields by tracking surface texture between reference and deformed images. Its non-contact implementation, full-field output and adjustable spatial resolution have led to increasing use in high-temperature deformation measurement [1,2,3,4].

High-temperature DIC is simultaneously affected by thermal radiation, speckle evolution, and changes in imaging geometry. Radiation saturation and speckle oxidation degrade grayscale correspondence, whereas refractive index fluctuations, focus drift, vibration, and out-of-plane motion introduce non-mechanical apparent deformation [3,4,5,6,7,8]. Recent experiments have further shown that oxidation-driven speckle degradation and thermally induced out-of-plane deformation can invalidate 2D-DIC measurements [9]. These findings establish that image quality and geometric stability can limit measurement validity, but they do not determine whether spatial field dispersion and condition-level scalar error respond to the same disturbances or can be assessed using the same endpoint.

Existing improvements to high-temperature DIC can be grouped into three broad categories. The first improves image visibility and speckle distinguishability through active monochromatic illumination, narrow-band filtering, high-temperature or projected speckles, and color channel discrimination [5,6,7,10,11,12,13]. The second suppresses frame-to-frame disturbances using grayscale averaging, grey-level correction, spatiotemporal DIC, or learning-based image restoration [14,15,16,17]. More recently, camera-noise-aware adaptive denoising combined with KAZE-feature-guided preregistration reduced thermally disturbed displacement errors and strain curve fluctuations [18]. A third category estimates thermally induced displacement and imaging drift using reference specimens, dual-channel configurations, or multispectral information [10,13,19,20,21,22]. Temperature changes in an imaging system can cause image translation and scale expansion, leading to systematic apparent displacement or strain errors [21]. Although these approaches improve image quality, speckle stability, displacement fluctuations, or thermally induced geometric errors, the relation between field stabilization and independently referenced condition-level scalar accuracy remains insufficiently resolved. High-temperature tests using Ncorr and an extensometer reference have also shown that strong DIC–reference correlations can coexist with speckle- and temperature-dependent condition-level discrepancies [23], further motivating separate evaluation endpoints. Without this distinction, parameter choices based primarily on field smoothness may be misinterpreted as evidence of quantitative accuracy, while systematic condition-level bias remains undetected. Previous high-temperature DIC studies have assessed measurement performance using several types of endpoints, including image similarity and speckle quality, temporal displacement or strain fluctuations, and agreement with synthetic ground truth or independent sensors. Image-domain quality and field-level repeatability characterize correspondence stability, but neither directly quantifies condition-level bias. Quantitative accuracy can be established only when DIC results are compared with known ground truth or an independent reference measurement. The distinction among these endpoints remains important when evaluating whether an image-processing method improves field appearance, scalar accuracy, or both.

Our work addresses this gap by distinguishing two endpoints that are often conflated in high-temperature 2D-DIC: strain field visualization stability and condition-level incremental strain accuracy. A dual-path framework evaluates these endpoints separately and tests whether improved field stability is accompanied by improved extensometer-referenced scalar accuracy. Controlled known-truth simulations are then used to distinguish the responses to local non-affine disturbance and global scale change, providing evidence for strain radius and feature selection. The framework is further assessed using a commercial monocular imaging system and an MA956 specimen subjected to open induction heating at 600–800 °C, with an independent extensometer reference and condition-level internal assessment. This distinction is important because field smoothness may otherwise be mistaken for evidence of quantitative accuracy while systematic scalar bias remains undetected. Synthetic data are used exclusively for mechanism verification and analysis design and are excluded from experimental data model fitting.

## 2. Materials and Methods

**Figure 1:**
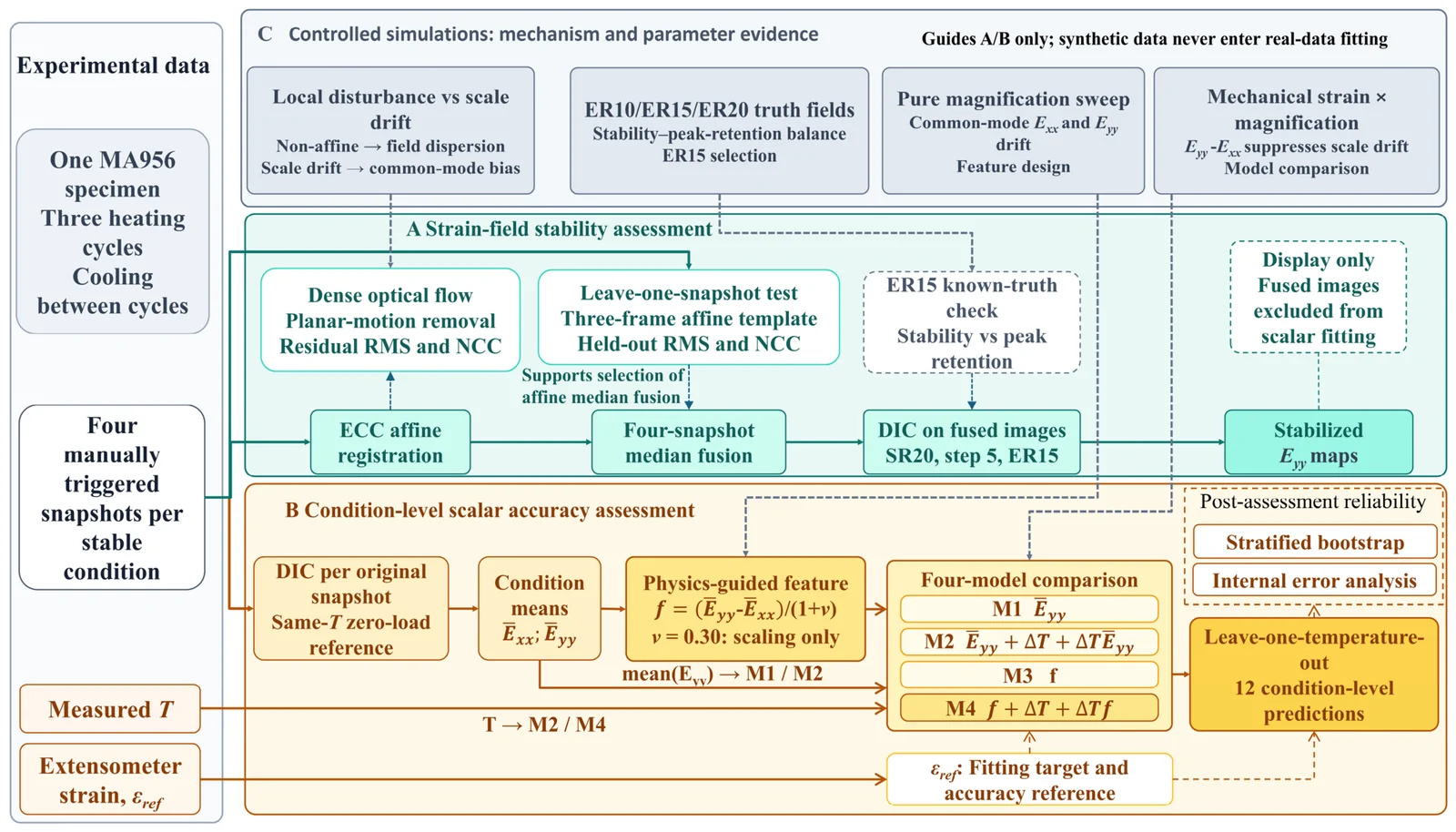
Study design for separate assessment of strain field visualization stability and condition-level incremental strain accuracy. Controlled simulations guide mechanism interpretation, ER15 selection, and common-mode feature construction without entering real-data fitting. Solid arrows denote experimental processing; dashed arrows denote simulation-based mechanism or method selection evidence.

Figure 1 summarizes the study workflow. The experimental data were analyzed along two separate paths. Section 2.3 and Section 2.5 assess strain field visualization stability through strain radius sensitivity analysis and multi-snapshot image processing, whereas Section 2.6 evaluates condition-level scalar accuracy against the extensometer reference. Controlled simulations with known ground truth are described in Section 2.4 and are used to distinguish the responses to local non-affine disturbance and global scale change and to support parameter and feature selection. Synthetic data do not enter the fitting or internal assessment of the experimental scalar models.

### 2.1. Experimental System and Specimen

**Figure 2:**
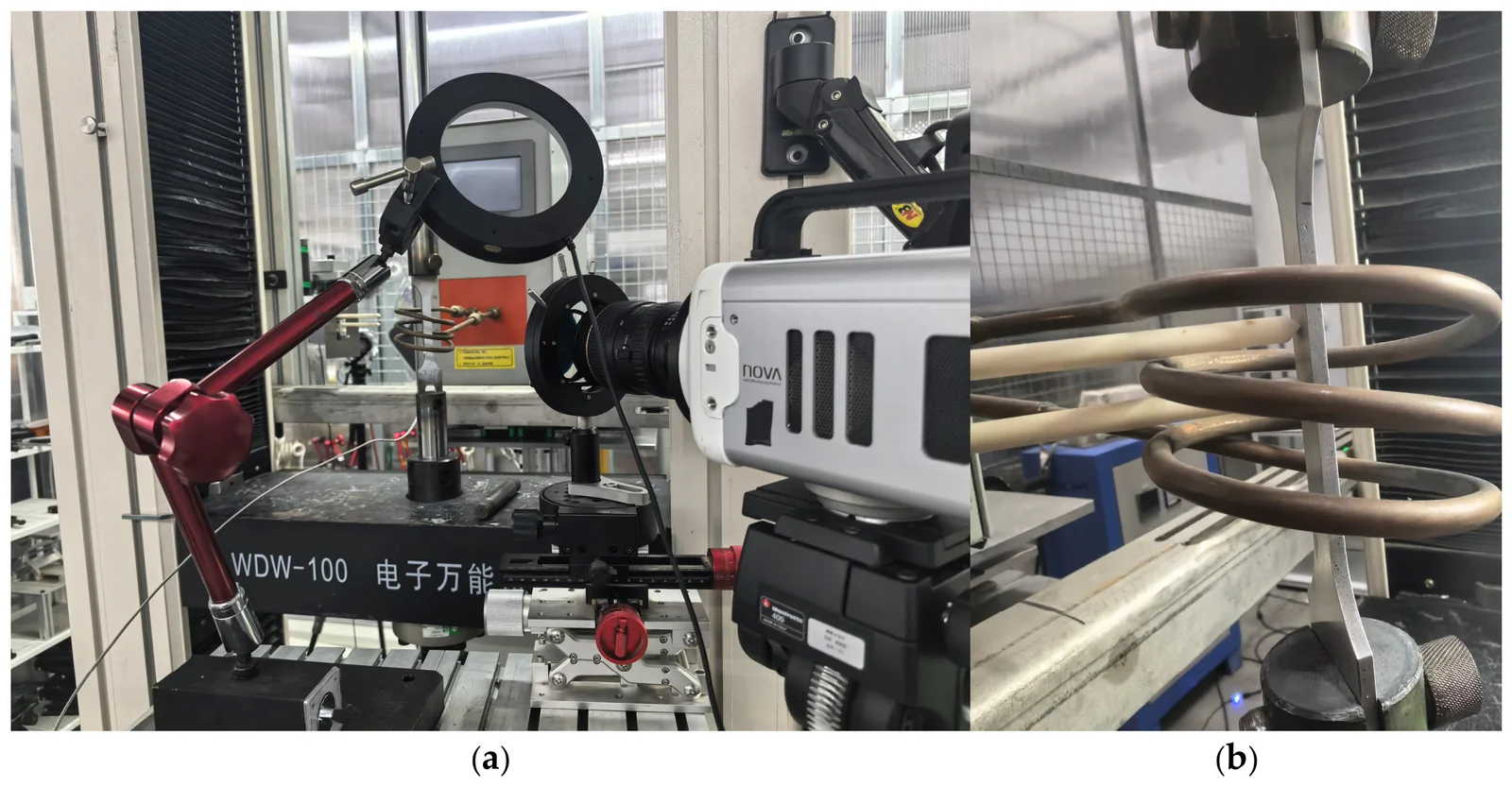
Experimental arrangement for high-temperature monocular DIC. (**a**) Relative positions of the high-speed camera, 100 mm macro lens, narrow-band filter, ring light, and WDW-100 testing machine; (**b**) close-up view of the MA956 specimen and induction coil. The Chinese text visible on the WDW-100 testing machine in panel (**a**) identifies the equipment as an electronic universal testing machine.

The experimental system comprised a WDW-100 universal testing machine, an open high-frequency induction heater, a Photron FASTCAM Nova S20 camera with a Tokina AT-X PRO 100 mm F2.8 Macro lens, a BP532-20K narrow-band filter, a white ring light, an M316 infrared pyrometer, and a YYHT-5 high-temperature extensometer with a 15 mm gauge length. Images were stored as 1024 × 1024-pixel, 8-bit BMP files at an exposure time of 0.008 s and a working distance of approximately 400 mm. At each stable condition, four snapshots were triggered individually through the PFV4 live interface rather than extracted from the 125 fps continuous stream. The camera and lens remained outside the heated region. The experimental arrangement is shown in Figure 2, and component specifications and acquisition settings are summarized in Table 1.

The lens aperture was set to F2.8 and camera gain was disabled throughout acquisition. Specimen edges were extracted from four room-temperature, zero-load images over *y* = 370–515 pixels. Using the nominal parallel-section width of 12.5 mm yielded a mean in-plane scale of 0.074205 mm/pixel (13.4761 pixel/mm). This scale was used only to convert displacement and spatial dimensions; it did not enter the dimensionless strain calculation. Because intrinsic camera and lens distortion calibrations were unavailable, the scale does not account for perspective or distortion.

Quantitative analysis used the same MA956 alloy specimen. The 600, 700, and 800 °C tests were conducted in three separate heating–loading cycles. After each cycle, the specimen was unloaded and cooled to room temperature before the next temperature cycle. Each temperature included nominal states of 0, 500, 1000, 1500, and 2000 µε. The 0 µε images at each temperature served as the corresponding DIC reference, and the four loaded states were used for quantitative modeling and assessment, giving 12 temperature load conditions. The resulting temperature–strain matrix is summarized in Table 2. The reported response is therefore incremental mechanical strain relative to the zero-load reference at the same temperature and excludes the total thermal strain accumulated from room temperature to the target temperature. Section 2.2 defines the four images and their role as technical replicates; they do not represent independent specimens or independent loading cycle replicates.

The specimen surface and speckle pattern were prepared as follows. A white high-temperature paint was first sprayed onto the specimen and allowed to dry. The coated specimen was then heated to 800 °C on the test platform so that the white layer underwent an oxidation-stabilization process in which it changed from white to yellow and then returned to white. A brown high-temperature paint was subsequently sprayed onto the oxidized white base to form a dark random speckle pattern. Images were acquired using the actual optical system and screened descriptively using mean grayscale, grayscale standard deviation, proportions of underexposed and saturated pixels, and Laplacian variance. Visual inspection was additionally used to confirm speckle coverage, sharpness, and continuity across the gauge section [24]. No retrospective exclusion threshold was imposed; all original images from the 12 quantitative conditions entered the analysis. This screening established image analyzability but did not replace extensometer-based error assessment.

**Figure 3:**
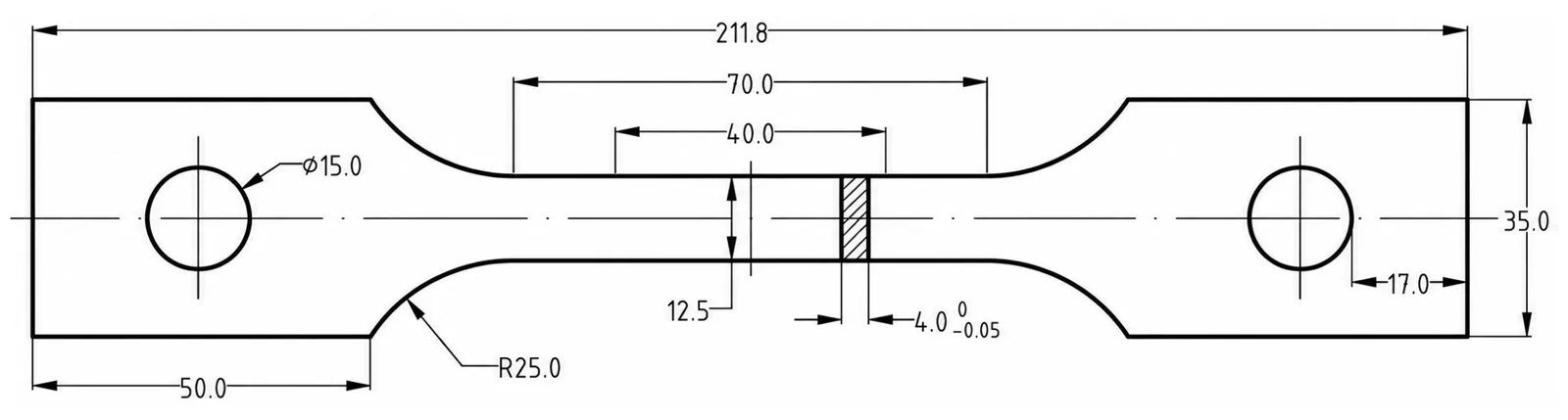
Geometry of the MA956 specimen (dimensions in mm).

The MA956 specimen had a total length of 211.8 mm, a 70.0 mm parallel section, a width of 12.5 mm, and a thickness of 4.0 mm (Figure 3). From the original testing machine records, the median absolute crosshead speed during the 15 s preceding the 12 loading peaks was 0.722 mm/min, and the corresponding median extensometer strain rate was 30.09 µε/s. These values characterize the recorded loading process rather than programmed machine settings. The extensometer reference was averaged from 25 s after each detected loading peak to 15 s before the subsequent peak; for the final loading state at each temperature, the averaging window ended 15 s before the end of the machine record. Across the 12 loaded conditions, these stable windows lasted 19.5–155.5 s (median, 93.9 s). The loading states were defined by extensometer strain, and no assumption of strictly elastic material behavior was used in fitting the experimental models.

### 2.2. Test Protocol and Temporal Correspondence

**Figure 4:**
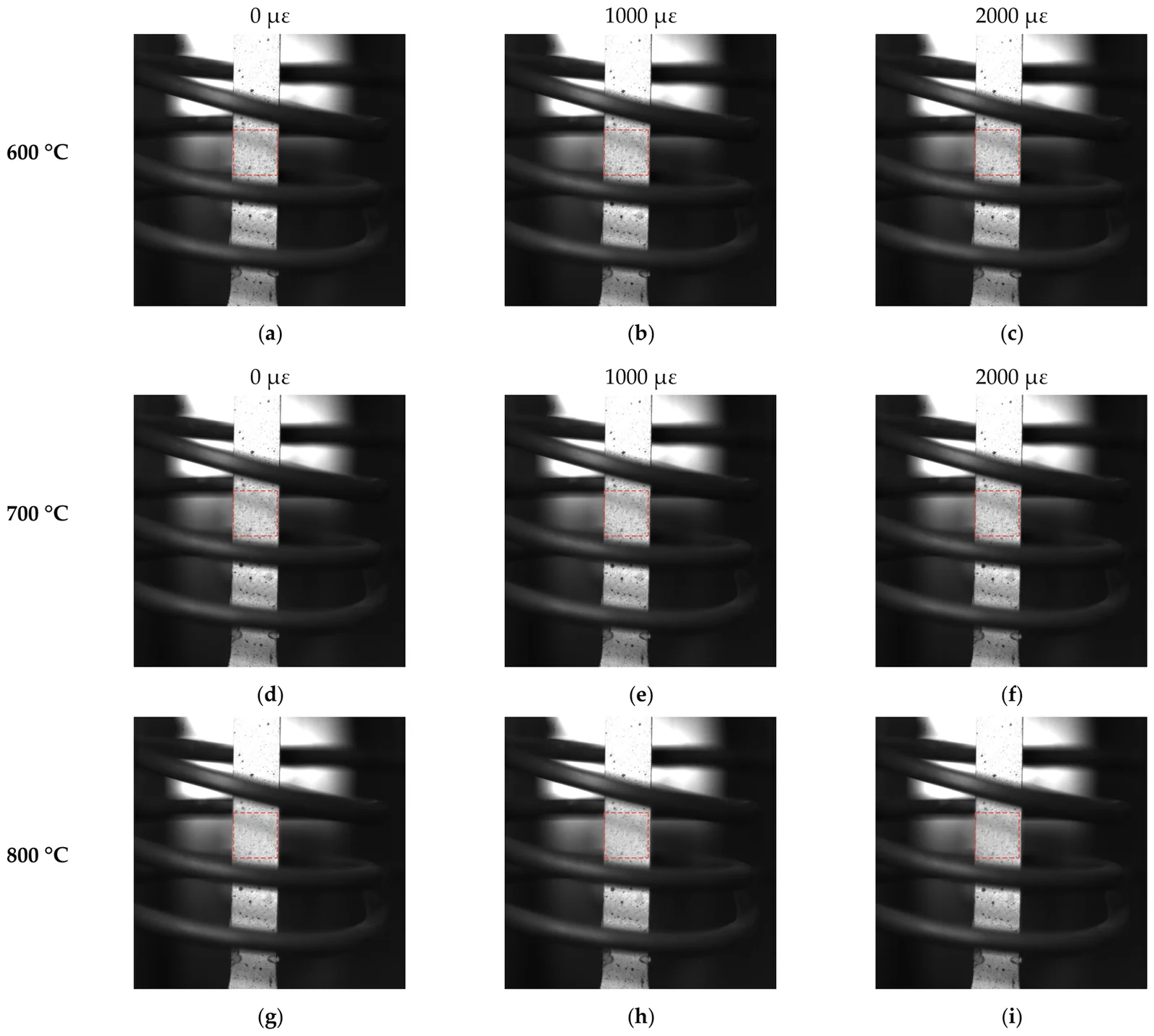
Representative raw images from the MA956 high-temperature tensile tests: (**a**–**c**) 600 °C at 0, 1000, and 2000 µε, respectively; (**d**–**f**) 700 °C at 0, 1000, and 2000 µε, respectively; and (**g**–**i**) 800 °C at 0, 1000, and 2000 µε, respectively. Each panel shows the first manually triggered snapshot from the corresponding stable condition. Red dashed boxes indicate the fixed DIC ROI. Images were only proportionally arranged and annotated; no registration, fusion, denoising, contrast enhancement, or grayscale remapping was applied.

At each target temperature, the operator separately triggered four snapshots through the PFV4 live interface after the temperature had overshot and returned to *T* ± 0.5 °C and after the extensometer had reached the target strain and a stable plateau. The 8-bit BMP images were separately triggered and separately exposed technical replicates, not independent specimen or cycle replicates. Across the 12 loaded conditions, the within-group range of matched image temperatures was 0.043–0.313 °C (median 0.129 °C), and the largest standard deviation in the ±2 s temperature windows was 0.243 °C. The extensometer plateau standard deviation ranged from 2.52 to 37.85 µε (median 19.20 µε). Conditions were confirmed during the experiment from the displayed temperature and strain values. The four image files for each loaded condition had NTFS last-write-time spans of 6–23 s (median, 7 s); however, these are file system save times rather than hardware camera timestamps and were not synchronized to the testing machine clock. Temporal correspondence was therefore established operationally from the stable displayed state and acquisition order, rather than through hardware-level timestamp alignment. Because snapshot intervals were not fixed, the 125 fps interface setting does not imply a four-frame acquisition frequency. For scalar analysis, loaded snapshot *k* was correlated with the corresponding zero-load snapshot acquired at the same temperature (*k* = 1–4). The four snapshot-level strain estimates were then averaged to obtain the condition-level value, with the mean extensometer reading over the stable plateau used as the reference strain. Representative raw images at 600, 700 and 800 °C are shown in Figure 4. The correspondence among image identifiers, temperature records, loading conditions, and extensometer references is provided in Supplementary Data S2. In each image, the fixed ROI lies within a continuous, unobstructed speckle region between adjacent coil turns, with no visible ROI occlusion or extensive intensity saturation.

### 2.3. Baseline 2D-DIC Analysis

DIC was performed using Ncorr [25] and a companion batch-processing program. The fixed ROI was [*x*_1_, *x*_2_, *y*_1_, *y*_2_] = [375, 540, 360, 530] pixels. A subset radius of 20 pixels, a grid spacing of 5 pixels, and a strain radius of 15 grid points (ER15) were used for the final analysis; strain radii of 10 and 20 grid points (ER10 and ER20, respectively) served as sensitivity settings. ER15 has a diameter of 31 strain grid nodes, corresponding to 150 pixels before boundary truncation. *E_xx_*, *E_xy_*, and *E_yy_* were calculated using the full Green–Lagrange formulation. Frame-level features were the means of valid ROI points. Spatial standard deviation and the difference between the 95th percentile (*P_95_*) and the median described overall dispersion and the positive high-value tail, respectively. A common validity mask was applied without manual point deletion [26,27,28]. The batch program handled ROI input, parameter assignment, frame-level statistics, and output export.

Monocular 2D-DIC assumes an approximately planar measured surface and a stable camera specimen pose. Because camera intrinsics and lens distortion were not calibrated, out-of-plane motion, perspective effects, and distortion remain incompletely quantified systematic error sources [29,30].

### 2.4. Controlled Synthetic Datasets and Mechanism Verification

Four classes of 512 × 512 pixel, 8-bit synthetic speckle images were constructed and analyzed using the same Ncorr parameters as the real data. The first class contained uniform, gradient, and localized concentration fields at low-, medium-, and high-disturbance levels, with four frames per state. These categories distinguish prescribed disturbance amplitudes and do not represent physical temperatures. Twenty-seven strain radius–field combinations were used to compare ER10, ER15, and ER20 in terms of field RMSE, spatial correlation, and peak retention.

The second class fixed *E_yy_* = 1000 µε and *E_xx_* = −300 µε and orthogonally combined a 0.030-pixel RMS local non-affine disturbance with a 300 parts-per-million (ppm; 10^−6^ relative scale) isotropic scale change to test whether field dispersion and ROI-mean bias could vary independently. The 0.030-pixel amplitude lies within the measured affine-removed residual RMS range of the real sequences (0.028–0.042 pixels), whereas 300 ppm represents a small-scale drift within the −600 to 600 ppm range used in the fourth simulation class. The third class imposed 11 pure magnification changes from −2000 to 2000 ppm and used *E*_app_ = (*M*^2^ − 1)/2 as the theoretical Green–Lagrange apparent strain to verify the biaxial common-mode response.

The fourth class fully combined mechanical strains of 500–2000 µε with magnification changes from −600 to 600 ppm, giving 20 conditions with four frames each. The ground-truth Poisson’s ratio was 0.30, and deformation was applied at fourfold oversampling before downsampling. This matrix compared the scale sensitivity of raw axial strain and the common-mode feature. Essential processing and controlled-simulation parameters are provided in Supplementary File S1. All four simulation classes were used only to verify algorithmic mechanisms and parameter choices and were excluded from MA956 model fitting.

### 2.5. Field Stability Assessment

Temperature-dependent disturbance diagnosis used two auxiliary MA956 sequences. The freely suspended sequence contained room-temperature, 600, 700, and approximately 798 °C states; the zero-load sequence used the 0 µε states at 600, 700, and 800 °C from the main tensile tests. Four technical replicate images at each state formed six snapshot pairs that shared original images. In a region extending 45 pixels beyond the ROI, a six-parameter two-dimensional affine transformation was first estimated by maximizing the enhanced correlation coefficient (ECC) criterion [31] (up to 300 iterations; convergence threshold 10^−8^). Bilinear interpolation with reflective boundary handling removed translation, rotation, shear, and first-order scale changes. Farnebäck dense optical flow [32] was then computed within the fixed ROI (pyr_scale = 0.5, levels = 4, winsize = 25, iterations = 5, poly_n = 7, and poly_sigma = 1.5). Residual planar components were removed separately from the horizontal and vertical flow fields by four iterations of robust least-squares fitting using a threshold of 3.5 × MAD. For each component, the median absolute deviation (MAD) was calculated from the corresponding planar fit residuals as $MAD\left(r\right)=median(\left|r_{i}-median\left(r\right)\right|)$, where *r_i_* denotes the planar fit residual at pixel *i*.

(1) $$R_{\mathrm{non}\text{-}\mathrm{aff}}=\sqrt{\frac{1}{N}\;\sum_{i=1}^{N}\;({u_{i}}^{2}+{v_{i}}^{2})}$$

where *u_i_* and *v_i_* are the horizontal and vertical residual displacement components at pixel *i*, respectively, and *N* is the number of valid pixels. The normalized cross-correlation coefficient (NCC) was calculated after affine registration. Concurrent increases in *R*_non-aff_ and reductions in NCC were interpreted as temperature-dependent non-rigid image disturbance rather than unique evidence of heat haze; illumination changes, speckle evolution, focus drift, vibration, and out-of-plane motion remain possible [15,16,19,20,33,34].

The strain field visualization path used ECC affine registration, bilinear resampling, and pixelwise median fusion of four images, followed by ER15 computation of *E_yy_* with a common 400–2600 µε color scale. Fused images did not enter the scalar model and were used only to display stable conditions; they were not used to infer transient, creep, or vibration strain histories.

To avoid evaluating an image against a template to which it contributed, one snapshot was held out in turn at each state and the remaining three formed the template-construction set. Pairwise similarities were first calculated only among the three training snapshots, and the medoid, defined as the snapshot with the smallest mean pairwise dissimilarity, was selected as the registration reference. The other two images were registered to the medoid using six-parameter ECC affine transformations, and the three registered images were fused by a pixelwise median to form an affine three-snapshot template. The held-out snapshot did not participate in medoid selection, transformation estimation, or template fusion; it was registered to the completed template only for residual RMS and NCC evaluation. Each snapshot was held out once. Four methods were compared: a raw single snapshot, an affine three-snapshot template, a non-affine-corrected single snapshot, and non-affine three-snapshot fusion. The 15 temperature load states, including zero-load states, yielded 60 held-out evaluations. This analysis characterizes processing consistency within shared acquisition groups and is not inferential evidence from independent specimens or loading cycles. Final display products used all four snapshots; the three-snapshot templates were used only for method selection.

### 2.6. Physics-Guided Scalar Modeling and Internal Validation

For scalar modeling, DIC was performed independently on the four original images, and frame-level features were averaged at the temperature load condition level. To motivate the axial-transverse feature, consider the superposition of uniaxial mechanical strain *ε* and an isotropic imaging-scale change. Under a first-order small-strain approximation, the observed axial strain is expressed as $E_{yy}^{obs}\approx \varepsilon +m$, whereas the observed transverse strain is $E_{xx}^{obs}\approx -v\varepsilon +m$, where *M* is the imposed isotropic linear image magnification factor and $m=\frac{\left(M^{2}-1\right)}{2}$ is the corresponding apparent Green–Lagrange strain. Subtracting the two expressions cancels the first-order common-mode scale term *m*, motivating the definition.

(2) $$f=\frac{\mathrm{mean}(E_{yy})-\mathrm{mean}(E_{xx})}{1+\nu }$$

where mean(*E_yy_*) and mean(*E_xx_*) are the ROI-mean axial and transverse DIC strains, respectively, and *ν* = 0.30. Under an idealized small-strain uniaxial elastic relation used only to motivate the feature, together with an isotropic scale disturbance, *f* approximately recovers the mechanical axial strain. Real measurements, however, also contain the material’s transverse response, lens distortion, local out-of-plane motion, temperature-dependent proportional error, and Ncorr bias. Therefore, *ν* is used only to construct a mechanically motivated feature; it is neither a fixed Poisson ratio correction nor estimated from these data. The transverse response may also include plastic or time-dependent material deformation; accordingly, the feature is interpreted only as a predictor after extensometer-based fitting, not as a direct elastic correction. The normalized temperature variable is defined as

(3) $$\Delta T=\frac{T-600\;{}^{\circ}C}{100\;{}^{\circ}C}$$

The calibrated strain is expressed as

(4) $${\hat{\varepsilon }}_{\mathrm{ref}}=c_{1}f+c_{2}+c_{3}\Delta Tf+c_{4}\Delta T$$

where *c*_1_–*c*_4_ are estimated by ordinary least squares with extensometer reference strain as the dependent variable. The interaction Δ*T f* represents a temperature-dependent proportional response, while Δ*T* represents a temperature-dependent offset. The available data show that applying a fixed Poisson’s ratio correction directly increases error. When *ν* is varied and the regression model is refitted, the fitted coefficient absorbs the feature rescaling; this sensitivity analysis therefore cannot be interpreted as identification of the material’s Poisson’s ratio.

In each leave-one-temperature-out fold, eight conditions from two temperature-associated cycles were used for fitting and four conditions from the remaining cycle were predicted, yielding 12 out-of-fold predictions. Holding out 700 °C is numerically an interpolation within the tested temperature range, whereas holding out 600 or 800 °C is a boundary extrapolation. However, because each temperature corresponds to one heating cycle, these folds cannot separate temperature effects from cycle-order or cumulative-thermal exposure effects. Metrics included bias, mean absolute error (MAE), root mean square error (RMSE), maximum absolute error, and *R*^2^. Conditions at the same temperature also reused the same zero-load reference images; the results are therefore used for within-specimen candidate model comparison and are not regarded as independent validation.

Four candidate models were prespecified and evaluated using identical leave-one-temperature-out folds. All were ordinary least-squares models with an intercept. M1 used mean(*E_yy_*); M2 used mean(*E_yy_*), Δ*T*, and Δ*T* × mean(*E_yy_*); M3 used only the common-mode suppression feature *f*; and M4 used *f*, Δ*T*, and Δ*T* × *f*, as in Equation (4). Here, Δ*T* = (*T* − 600)/100 was calculated from the measured condition-level temperature rather than the nominal temperature label. This comparison separately tests the contributions of temperature information, the common-mode feature, and their combination. Because model comparison and performance assessment used the same 12-condition dataset, the lowest RMSE is interpreted only as an exploratory internal comparison and not as independent performance after model locking.

Each M4 fold estimated four parameters from only eight conditions. A stratified nonparametric bootstrap was therefore performed by sampling the four load conditions with replacement within each training temperature, rejecting rank-deficient resamples until 5000 valid replicates were obtained (random seed 20260809). We report the column-scaled design matrix condition number, the bootstrap RMSE distribution, and the 2.5–97.5% bootstrap percentile interval of fitted predictions. These are model instability intervals that quantify sensitivity to the available training conditions; they are not independent prediction intervals, coverage guarantees, or measures of uncertainty from model selection, shared reference images, independent specimens, or heating cycles.

Use of AI-Assisted Tools: OpenAI Codex was used to assist with the organization and debugging of author-developed analysis scripts, figure layout, and cross-checking of computational procedures, parameter settings, numerical results, and figure content against the original data. It was not used to acquire or generate experimental data, define the scientific methodology, or make final analytical decisions. All AI-assisted outputs were reviewed and verified by the authors.

## 3. Results

### 3.1. Non-Rigid Disturbance Diagnosis and Multi-Snapshot Stabilization

**Figure 5:**
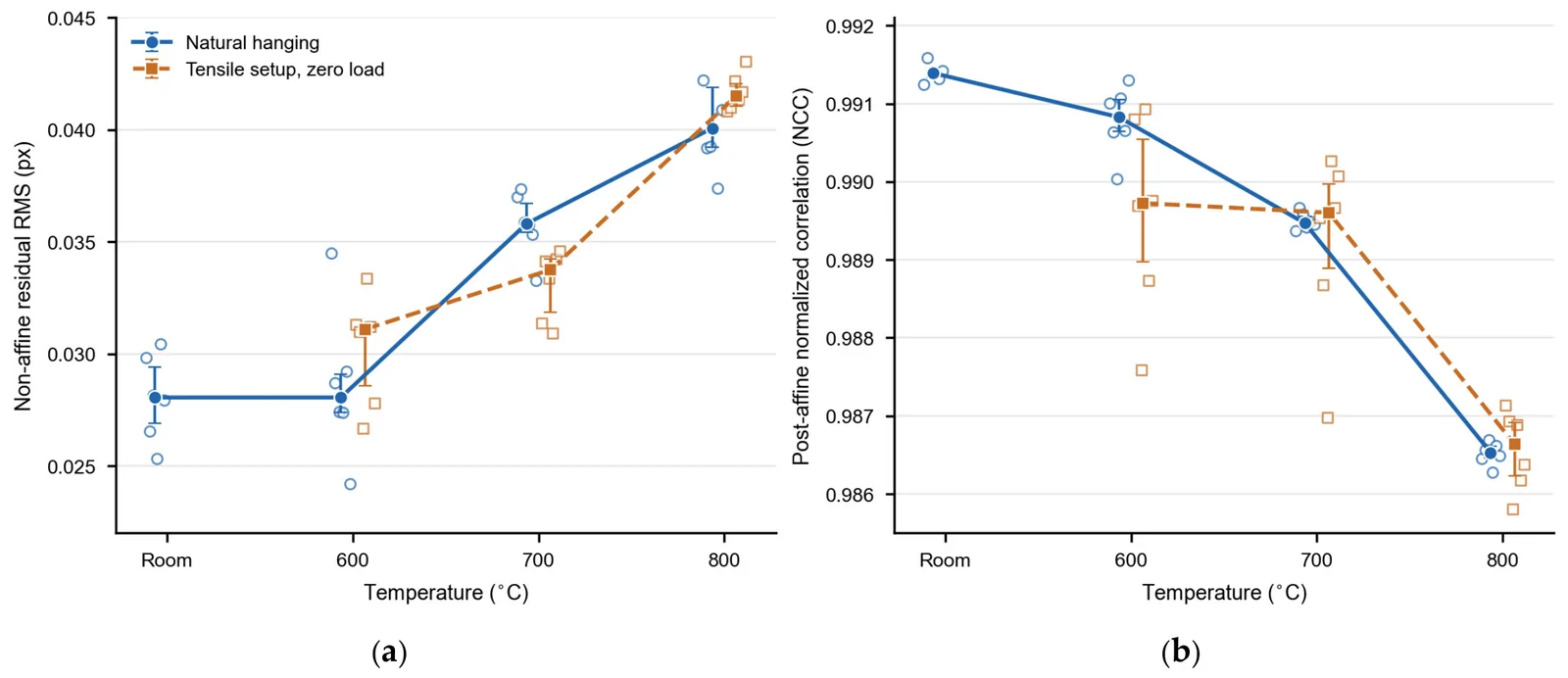
Temperature-dependent image-disturbance diagnostics: (**a**) non-affine residual displacement RMS after removal of the global affine transformation and residual planar flow; (**b**) post-affine normalized cross-correlation (NCC) within the fixed ROI. Dense optical flow was calculated after affine registration, and displacement-magnitude RMS was evaluated using Equation (1). Points denote within-state snapshot pairs, whereas the central markers and error bars denote the median and interquartile range, respectively.

After removal of global affine motion, both sequences showed greater non-affine residual displacement and lower image similarity at higher temperatures (Table 3 and Figure 5). In the freely suspended sequence, the median residual RMS increased by 42.9%, from 0.02805 pixels at room temperature to 0.04007 pixels at approximately 798 °C, while the median NCC decreased from 0.99139 to 0.98653. In the zero-load tensile sequence, the median residual RMS increased by 33.5%, from 0.03109 pixels at 600 °C to 0.04152 pixels at 800 °C, while the median NCC decreased from 0.98972 to 0.98663. Each temperature state comprised six pairwise comparisons constructed from four original snapshots; these comparisons were not statistically independent and were used only to describe temperature-related trends. Residual motion after affine correction indicates local image instability that cannot be explained by low-order global motion alone. The pattern is consistent with temperature-dependent refractive disturbance but does not establish heat haze as the unique cause.

**Figure 6:**
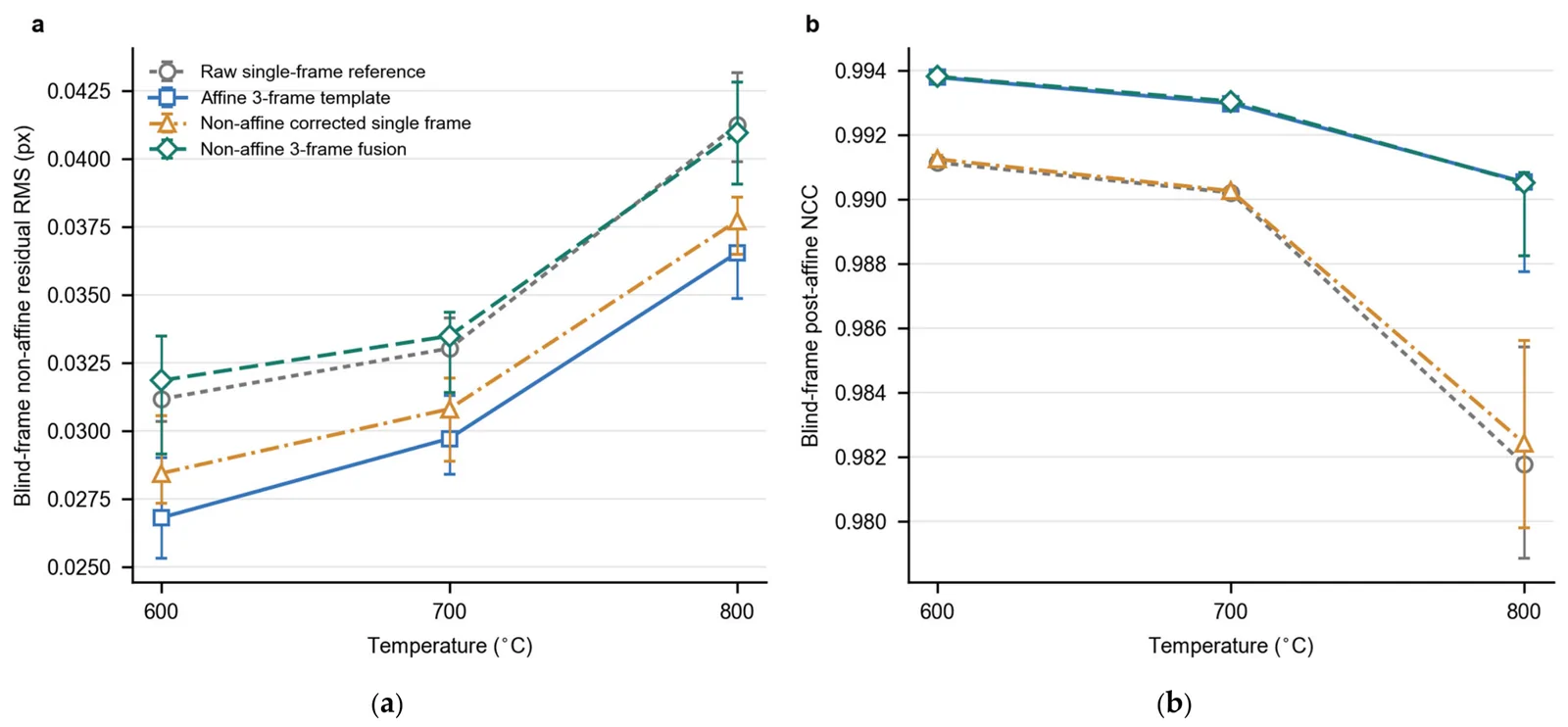
Paired held-out-snapshot evaluation of multi-snapshot stabilization methods: (**a**) blind-frame non-affine residual displacement RMS; (**b**) blind-frame post-affine normalized cross-correlation (NCC). For each evaluation, the other three snapshots from the same state formed the template, and the excluded snapshot was used to calculate the residual RMS and NCC. The shared technical-replicate images do not represent independent experimental replication. Curves overlap where methods yield identical or nearly identical median values; distinct marker shapes and line styles are used to distinguish the methods.

Relative to a raw single snapshot, the affine three-snapshot template reduced median held-out-snapshot residual RMS by 14.0%, 10.0%, and 11.4% at 600, 700, and 800 °C, respectively. Across the 60 paired technical evaluations, the affine template yielded lower RMS in 57 cases (57/60, 95.0%) and higher NCC in all 60 cases (60/60, 100%). A non-affine-corrected single snapshot yielded lower RMS in 56 cases (56/60, 93.3%), but the median RMS reductions at the three temperatures were only 8.8%, 6.7%, and 8.6%, all smaller than those achieved by the affine template (Table 4 and Figure 6). These proportions describe consistency of paired improvement on the same held-out snapshots; they are not success probabilities from independent experiments.

Non-affine three-frame fusion improved NCC in all 60 paired evaluations but reduced RMS in only 31 cases (31/60, 51.7%). Relative to the raw single snapshot, its median RMS increased by 2.18% and 1.38% at 600 and 700 °C and decreased by only 0.68% at 800 °C (Table 4). Improved grayscale similarity therefore did not consistently correspond to reduced geometric residual displacement. On this basis, affine four-snapshot fusion was retained for strain field visualization, whereas non-affine optical flow fusion was excluded from the scalar measurement path.

### 3.2. Strain Radius Sensitivity Using Real and Synthetic Evidence

This section first evaluates changes in field stability in the uniform gauge section of the real MA956 specimen and then evaluates spatial error and peak retention using synthetic fields with known ground truth, thereby avoiding parameter selection based solely on visually smoother maps. Increasing the strain radius from 10 to 15 grid points reduced the mean spatial standard deviation of loaded strain fields from 554.7 to 428.8 µε (22.7%) and reduced the mean upper-tail span (*P*_95_ minus median) from 814.3 to 678.9 µε (16.6%). For same-load snapshot pairs, the upper-tail span decreased from 254.0 to 138.3 µε (45.6%), while the standard deviation of the four frame-level mean strains changed only from 94.11 to 93.91 µε (0.2%). The ER15 leave-one-temperature-out RMSE was 76.27 µε, close to the ER10 value of 75.53 µε (Table 5). ER15 therefore primarily reduced local gradient noise and non-physical high-value tails without materially changing condition-level repeatability or scalar temperature-held-out performance.

**Figure 7:**
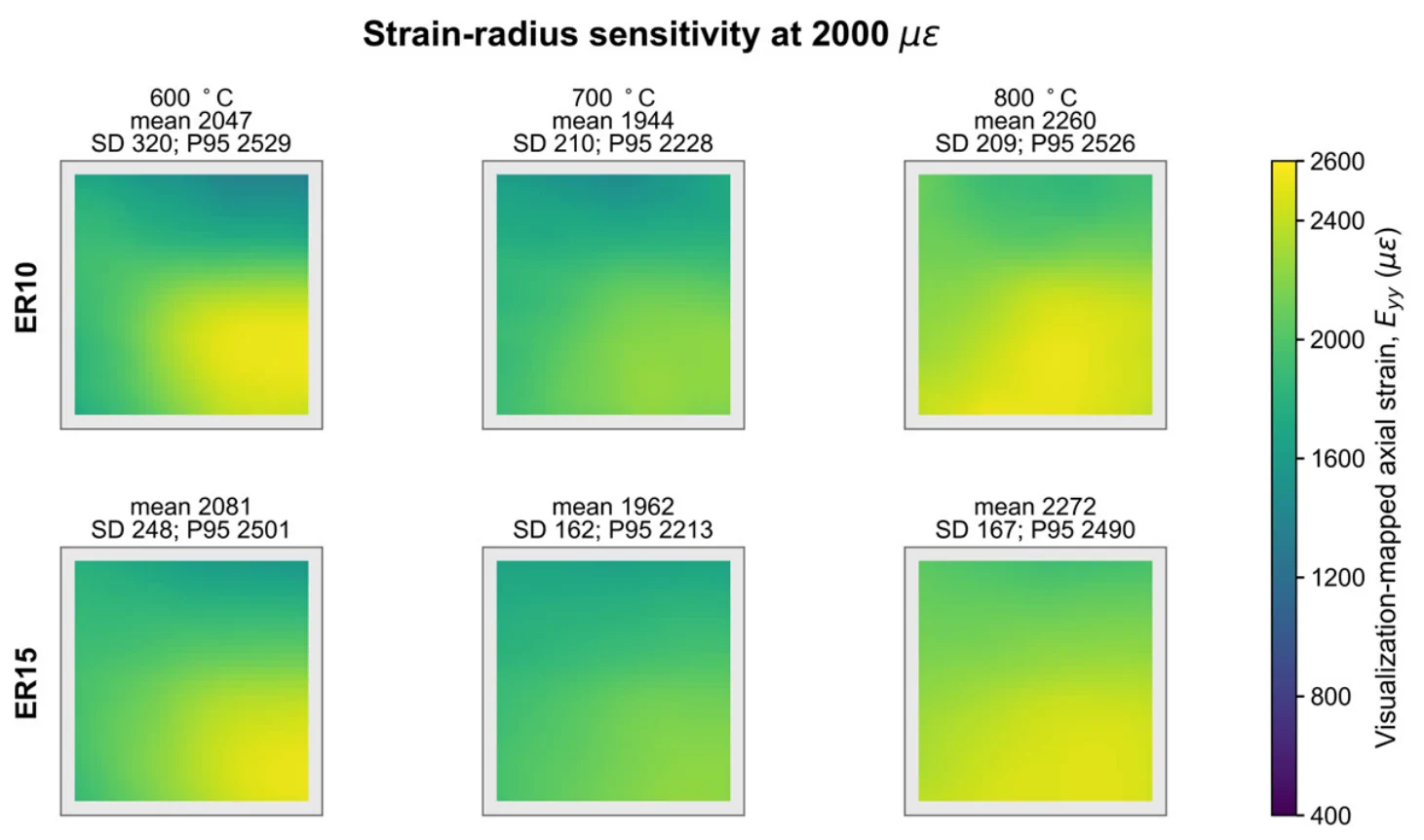
Strain radius sensitivity of affine-registered, four-snapshot median image templates at 2000 µε. The ER10 and ER15 panels show Ncorr-derived axial Green–Lagrange strain fields *E_yy_* using a common color scale. Mean, spatial standard deviation (SD), and *P*_95_ are reported for each field. These statistics quantify spatial smoothing and do not constitute pixelwise accuracy validation.

At 2000 µε, the spatial standard deviation of the affine-fused ER15 strain field was lower than that of ER10 at all temperatures: 320 to 248 µε at 600 °C, 210 to 162 µε at 700 °C, and 209 to 167 µε at 800 °C (Figure 7). The corresponding changes in mean strain were below 35 µε. Reduced spatial dispersion was accompanied by a small decrease in *P*_95_, reflecting the expected trade-off between noise suppression and peak sensitivity. ER15 was therefore selected for visualization of the uniform gauge section, with ER10 retained as a sensitivity reference rather than an alternative ground truth.

The known-truth synthetic results reproduced the smoothing peak trade-off observed in the real uniform gauge section (Table 6). ER10 had the highest overall field RMSE (290.3 µε) but the highest localized field *P*_95_ peak retention ratio (85.0%). ER20 reduced overall field RMSE to 188.0 µε but also reduced localized field *P*_95_ peak retention to 68.2%, indicating pronounced over-smoothing. ER15 yielded an overall field RMSE of 220.1 µε, spatial correlations of 0.960 and 0.874 for the gradient and localized fields, respectively, and a localized field *P*_95_ peak retention ratio of 76.6%. It was therefore selected as a compromise for the uniform gauge section.

**Figure 8:**
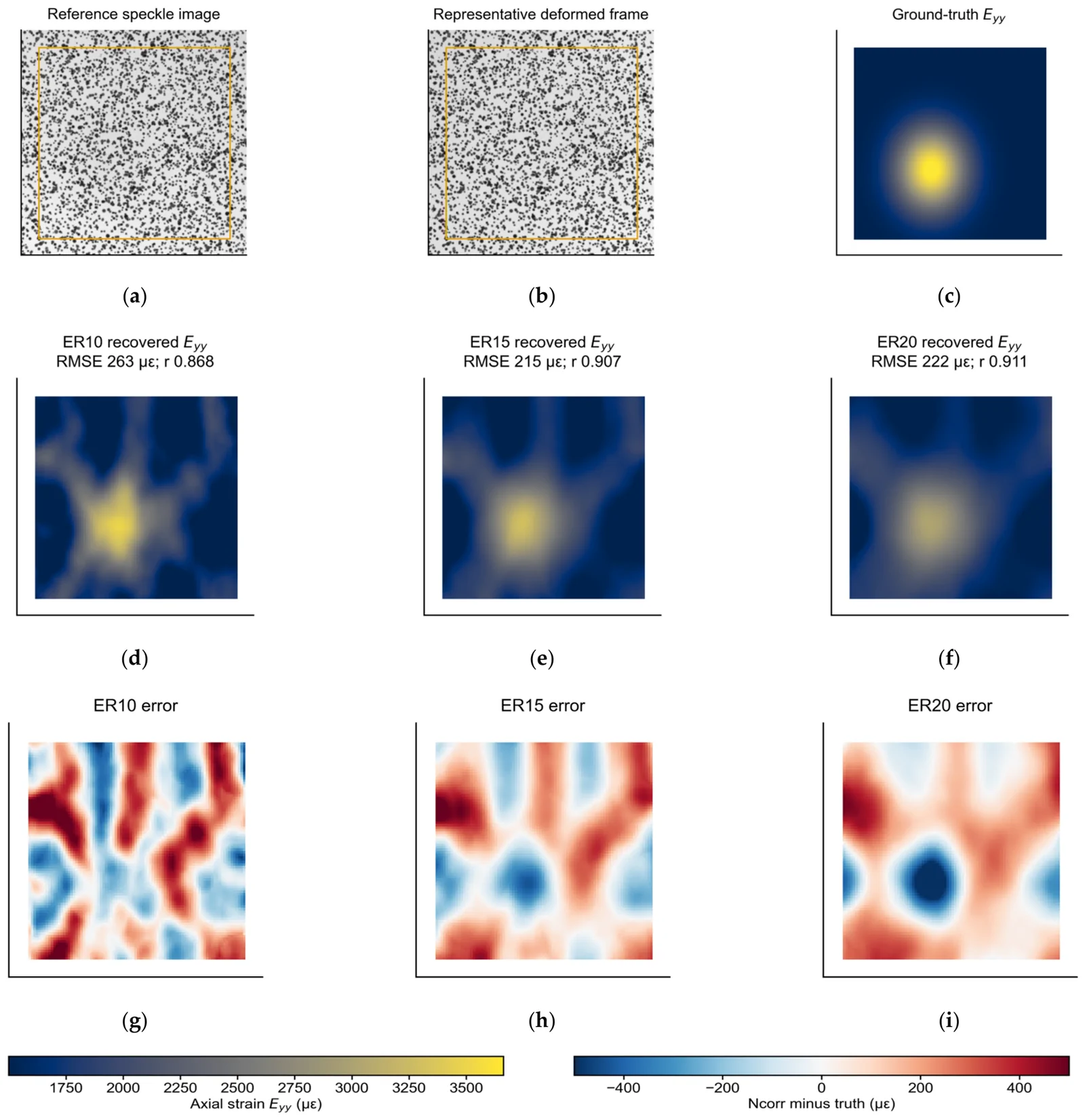
Representative known-truth validation of Ncorr strain reconstruction for a localized-concentration field at the high disturbance level. (**a**) Reference speckle image; (**b**) representative deformed image with a prescribed peak axial strain of 1500 µε; (**c**) prescribed axial strain ground truth; (**d**–**f**) Ncorr-reconstructed axial strain fields obtained from four-snapshot pixelwise-median image templates using ER10, ER15, and ER20, respectively, with a subset radius of 20 pixels and a grid spacing of 5 pixels; (**g**–**i**) corresponding reconstruction error fields, defined as the Ncorr result minus the ground truth. Panels (**c**–**f**) share the same strain scale, whereas panels (**g**–**i**) share the same symmetric error scale. This representative case illustrates the trade-off between spatial smoothing and peak retention; selection of ER15 was based on all 27 strain radius–field combinations summarized in Table 6.

Figure 8 shows representative strain fields, whereas ER15 was selected based on the aggregate evaluation of 27 radius–field combinations comprising three strain radii and nine synthetic strain fields (Table 6).

**Figure 9:**
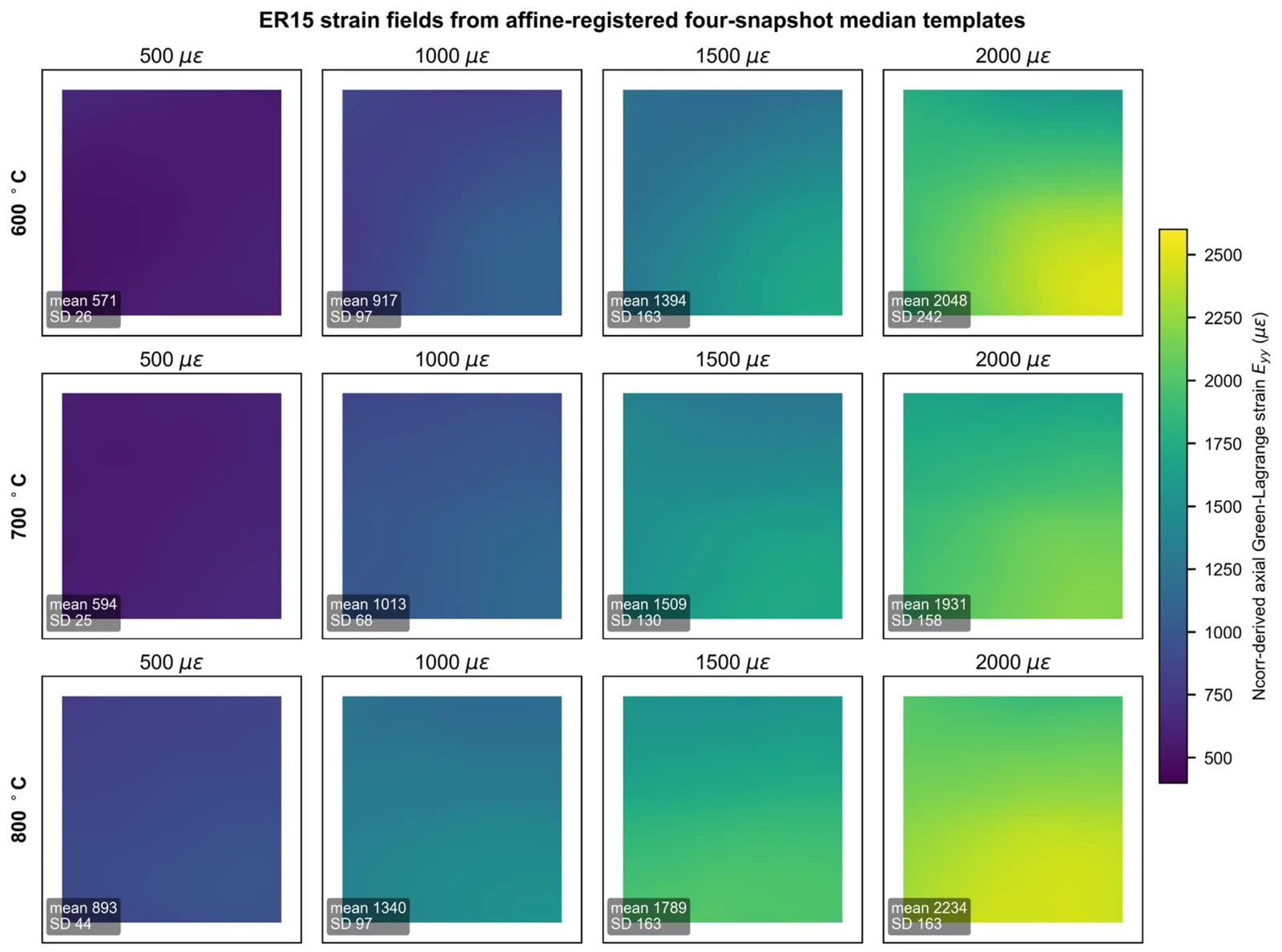
Ncorr-derived ER15 axial Green–Lagrange strain fields obtained from affine-registered, pixelwise four-snapshot median image templates. Rows correspond to 600, 700, and 800 °C, and columns correspond to nominal incremental strain levels of 500, 1000, 1500, and 2000 µε. All panels use the same 400–2600 µε color scale. No extensometer-based pointwise calibration or remapping was applied. The fields are presented only to compare spatial patterns and were excluded from condition-level scalar model fitting.

Figure 9 presents the Ncorr-derived ER15 axial Green–Lagrange strain fields obtained from affine-registered, pixelwise four-snapshot median image templates using a common color scale of 400–2600 µε. Field means increased with load, but temperature-dependent offsets persisted despite visually smooth spatial distributions. This contrast shows that field smoothness alone is insufficient to establish condition-level scalar accuracy, which must be evaluated against the extensometer reference.

### 3.3. Controlled Simulation I: Independent Variation in Field Stability and Scalar Error

**Figure 10:**
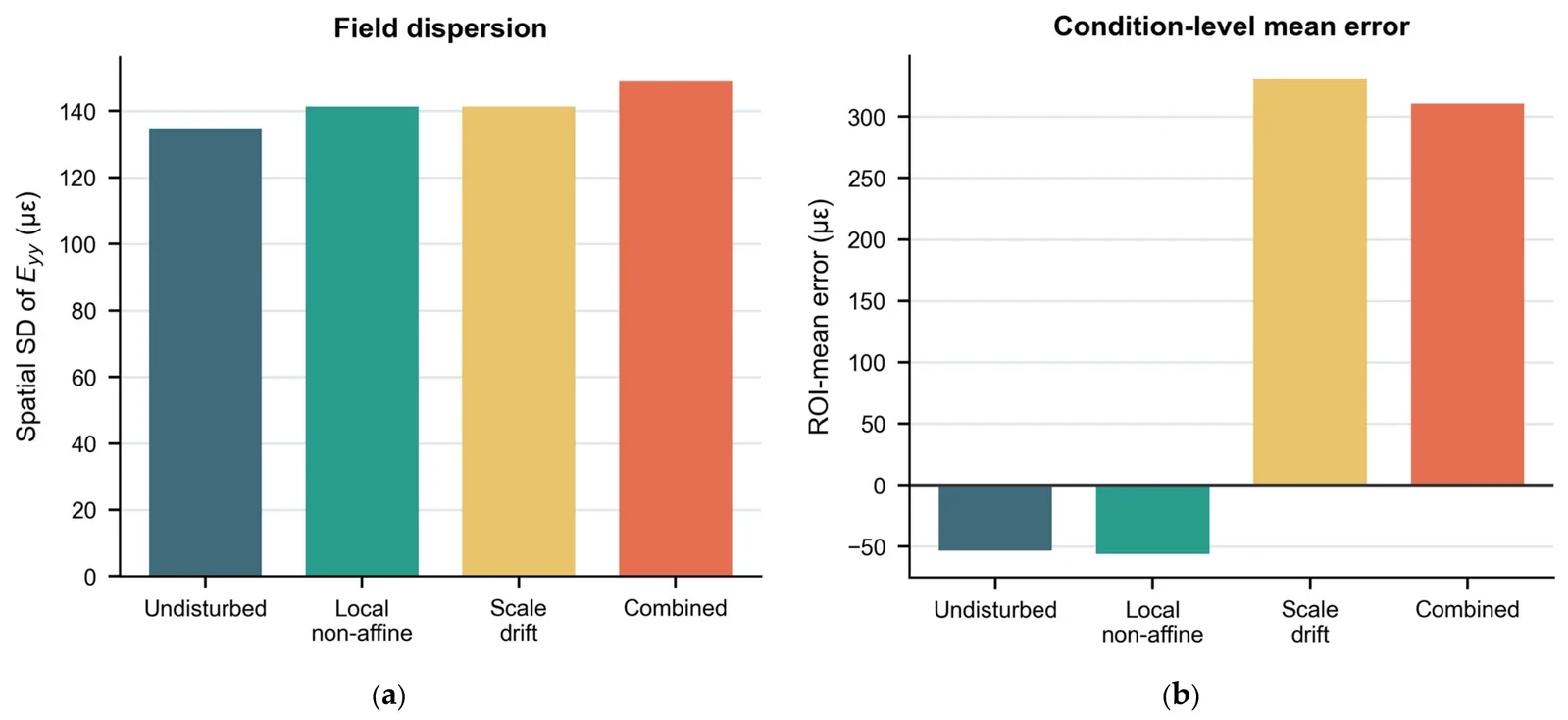
Controlled decoupling of field dispersion and ROI-mean error under independently imposed local non-affine disturbance and isotropic scale change. (**a**) Spatial standard deviation of the recovered axial strain, (E_{yy}), under the undisturbed, local non-affine, scale-drift, and combined-disturbance conditions; (**b**) corresponding condition-level ROI-mean axial-strain error relative to the known ground truth. All conditions share a mechanical axial-strain ground truth of 1000 µε.

With mechanical ground truth held constant, adding only the local non-affine disturbance increased the single-frame axial strain spatial standard deviation by 34.68 µε, whereas the increase after four-frame median fusion was only 6.61 µε; the fused scalar mean changed by −2.58 µε relative to the undisturbed condition. In contrast, adding only a 300 ppm isotropic scale change increased the fused-field spatial standard deviation by only 6.66 µε but shifted the Ncorr-derived fused ROI mean by 383.71 µε relative to the undisturbed condition. The 383.71 µε value is the numerical response after image interpolation, correlation, finite subset processing, and strain estimation; it is not obtained by directly equating 300 ppm with theoretical strain. The separate pure-scale sweep in Section 3.4 evaluates agreement with the theoretical Green–Lagrange response. Combining both disturbances produced both elevated spatial dispersion and an approximately 311 µε scalar bias (Table 7 and Figure 10). Thus, a smoother field does not establish a more accurate mean, and a stable mean does not exclude local field error.

### 3.4. Controlled Simulation II: Magnification-Induced Apparent Strain and the Common-Mode Feature

**Figure 11:**
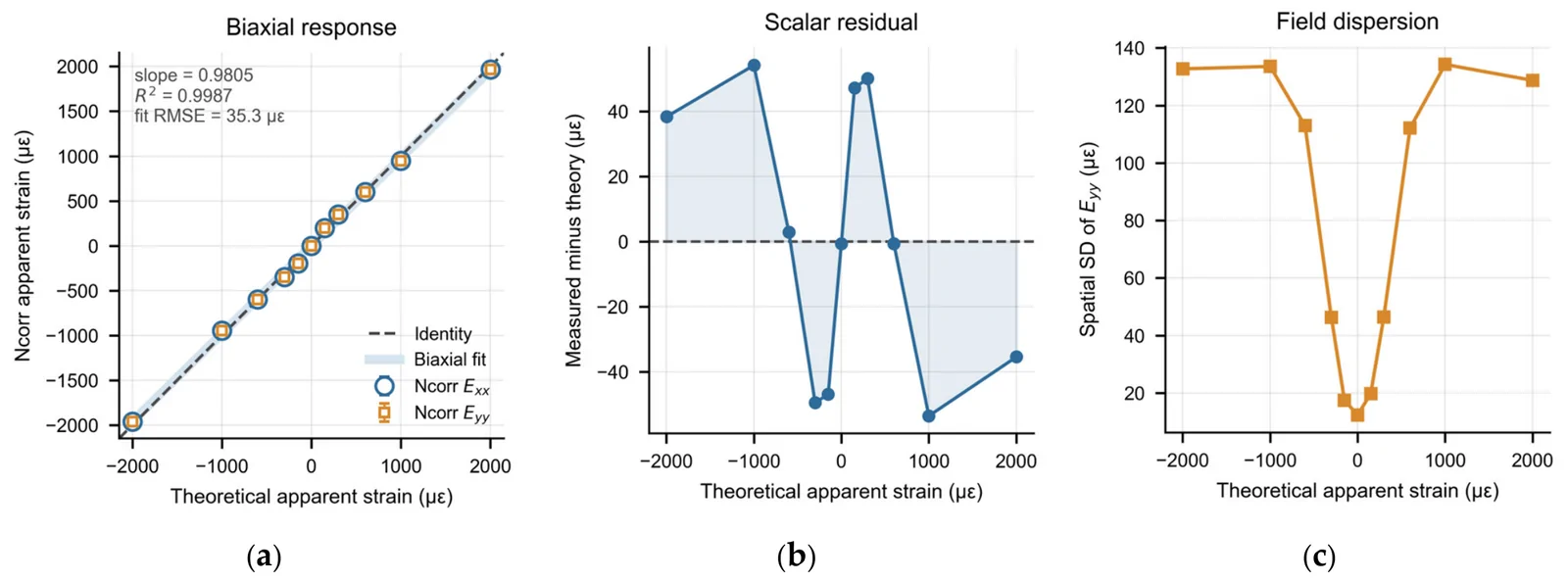
Ncorr-derived apparent strains induced by pure magnification changes in controlled synthetic images. (**a**) Biaxial response compared with Green–Lagrange theory; (**b**) scalar residuals; (**c**) spatial dispersion of the reconstructed strain fields.

Pure magnification changes produced approximately equal apparent strains in *E_xx_* and *E_yy_*. Across 11 scale conditions, linear fitting of the biaxial mean against the Green–Lagrange theory yielded a slope of 0.9805, an intercept of 0.57 µε, *R*^2^ = 0.9987, and an RMSE of 35.35 µε (Table 8 and Figure 11). Small magnification changes caused by out-of-plane motion or focal drift can therefore appear as approximately isotropic common-mode strain in monocular DIC. This result establishes a plausible mechanism but cannot be used to infer out-of-plane displacement in the real experiment.

**Figure 12:**
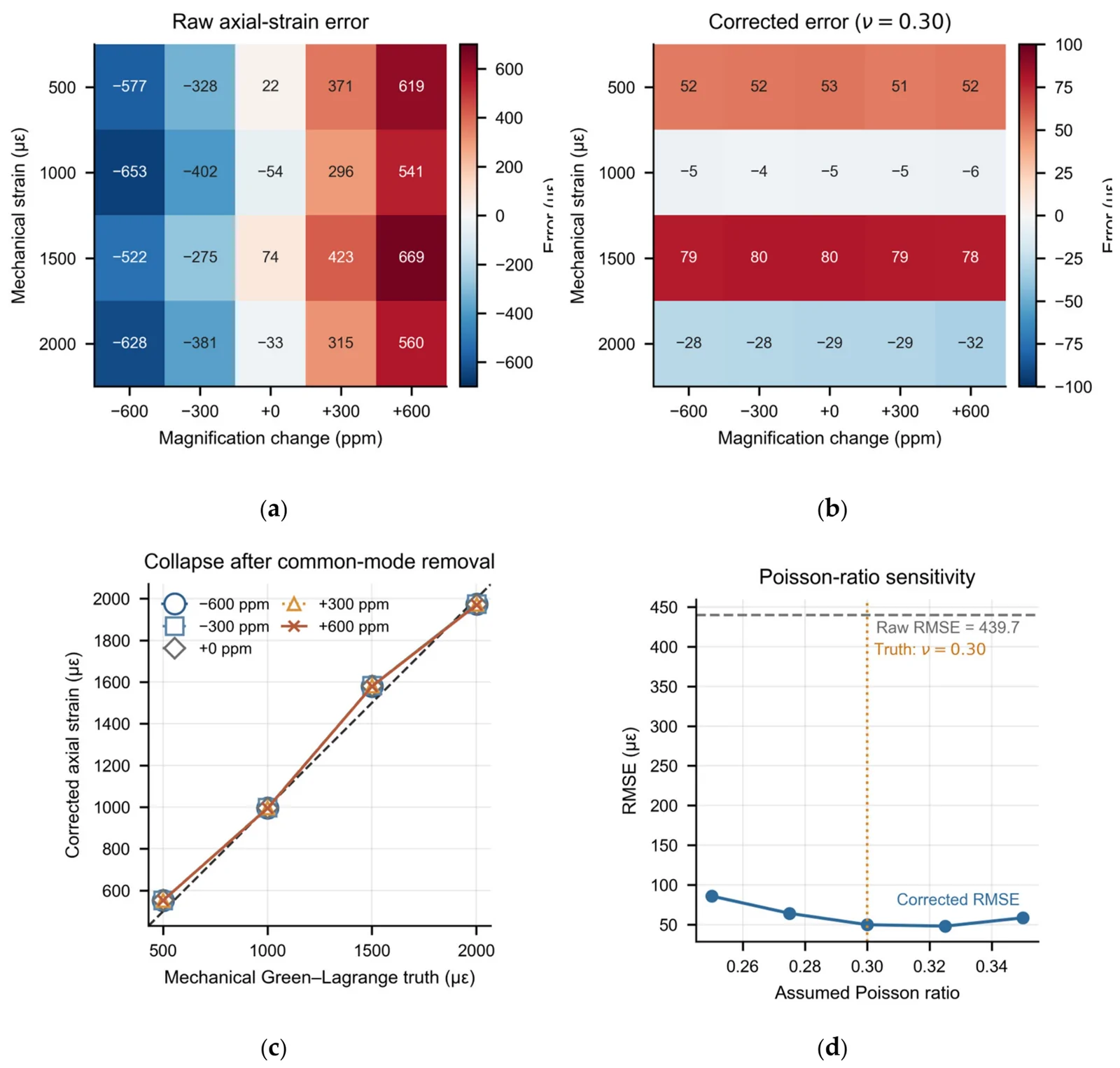
Controlled mechanical-strain-by-magnification simulation. (**a**) Raw axial error; (**b**) common-mode-suppressed error at *ν* = 0.30; (**c**) convergence across magnification conditions; (**d**) sensitivity to the assumed Poisson’s ratio.

Across the 20 fully crossed combinations of mechanical strain and ±600 ppm magnification change, the raw axial strain RMSE was 439.72 µε and the mean axial span across magnifications at a fixed mechanical load was 1192.34 µε. Common-mode suppression using *ν* = 0.30 reduced RMSE to 49.81 µε, an 88.67% reduction, and reduced the mean span to 2.64 µε (Table 8 and Figure 12). Changing the assumed *ν* changed the direct correction error: RMSE ranged from 47.998 to 85.904 µε for *ν* = 0.25–0.35. This variation reflects sensitivity to the feature assumption rather than measurement of the real material’s Poisson’s ratio.

### 3.5. Temperature-Conditioned Scalar Model and Leave-One-Temperature-Out Internal Assessment

Fitting all 12 ER15 conditions yielded *c*_1_ = 0.2747470, *c*_2_ = 393.2535, *c*_3_ = 0.00597630, and *c*_4_ = −235.3881, with an in-sample RMSE of 54.09 µε. These coefficients depend on the optical system, ROI, analysis parameters, and temperature normalization used here and are not material constants. Because candidate model comparison and internal assessment used the same dataset, the RMSE values below are interpreted as exploratory results.

**Figure 13:**
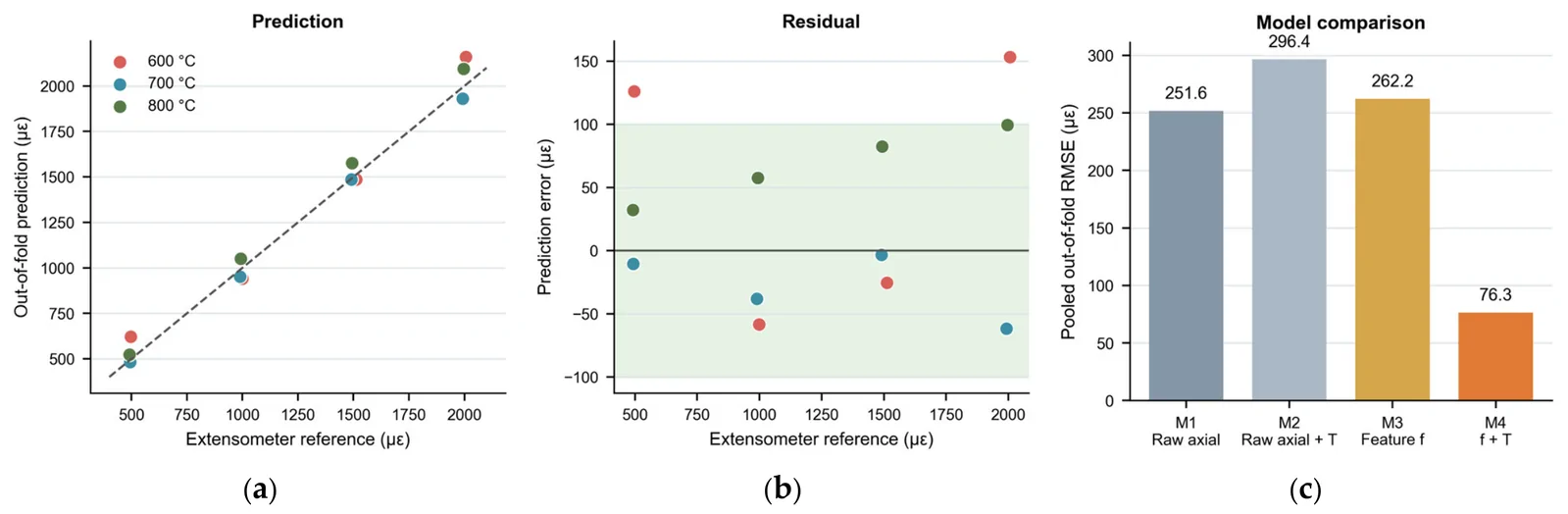
ER15 leave-one-temperature-out internal assessment of four prespecified models. (**a**) Out-of-fold predictions from M4 versus extensometer references; (**b**) M4 out-of-fold residuals by temperature, with the light-green band indicating ±100 µε; (**c**) pooled out-of-fold RMSE for M1 = mean(*E_yy_*), M2 = mean(*E_yy_*) + Δ*T* + Δ*T* × mean(*E_yy_*), M3 = *f*, and M4 = *f* + Δ*T* + Δ*T* × *f*, evaluated on the same 12 conditions using identical folds.

Table 9 and Figure 13 summarize the exploratory leave-one-temperature-out results. RMSE was lowest when 700 °C was held out (36.73 µε). Holding out 800 °C yielded an RMSE of 72.51 µε; all four residuals were positive, with a maximum absolute error of 99.42 µε. Errors were greatest when 600 °C was held out, with an RMSE of 104.14 µε and a maximum absolute error of 153.12 µε. Because each held-out temperature contained only four conditions, fold-specific *R*^2^ values are not reported; *R*^2^ was calculated only after pooling all 12 out-of-fold predictions. These comparisons do not establish performance beyond the tested range or across specimens.

The out-of-fold RMSE values of the four prespecified candidate models were 251.65, 296.40, 262.23, and 76.27 µε, respectively (Figure 13). Neither the temperature terms nor feature *f* alone improved on the baseline. In the present exploratory internal comparison, their combination reduced RMSE by 69.7% relative to the baseline and outperformed the other prespecified models. This is not yet independent performance evidence after model locking. Direct use of uncalibrated *f* yielded an RMSE of 3047.03 µε. The ER10 temperature-conditioned model yielded an RMSE of 75.53 µε, further showing that the common-mode feature must be jointly fitted to the extensometer reference and temperature, whereas ER15 primarily improves field display. Condition-level inputs, extensometer references, and ER10/ER15 out-of-fold predictions and residuals are provided in Supplementary Data S2.

**Figure 14:**
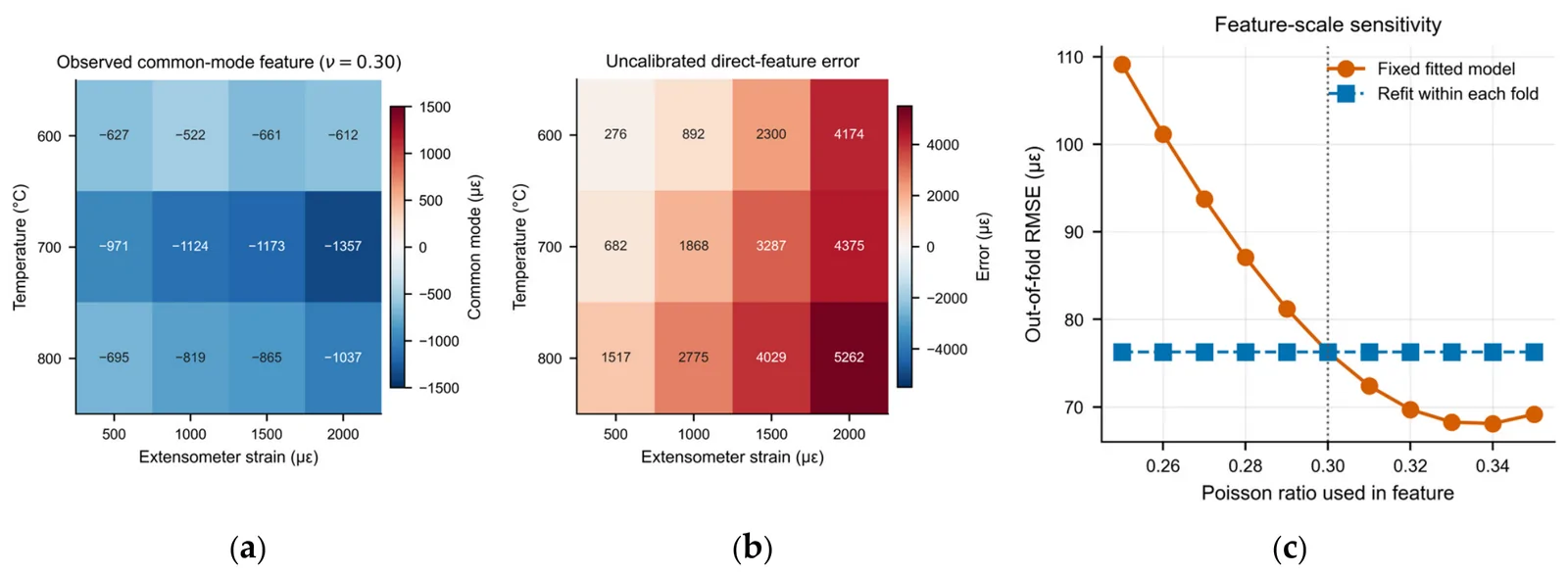
Behavior and applicability limits of the common-mode suppression feature in the experimental MA956 data. (**a**) Observed common-mode response calculated using *ν* = 0.30; (**b**) prediction error when the difference feature was applied directly without extensometer-based calibration; (**c**) sensitivity to the assumed Poisson’s ratio for a fixed fitted model and for models refitted within each validation fold. Synthetic data were excluded from all model fitting.

Figure 14 examines the behavior and applicability limits of the common-mode suppression feature in the experimental MA956 data. The observed common-mode response calculated using *ν* = 0.30 is shown in Figure 14a. Direct application of the difference feature without extensometer-based calibration produced systematic condition-dependent errors (Figure 14b), indicating that common-mode suppression alone does not provide an absolute strain estimate. Figure 14c compares sensitivity to the assumed Poisson’s ratio when the fitted model was held fixed and when it was refitted within each validation fold. The difference between these analyses shows that model fitting can absorb part of the apparent Poisson-ratio sensitivity. The feature should therefore be interpreted as a physics-guided predictor rather than a calibration-free correction formula.

Model stability analysis further qualifies the point estimates. When 600, 700, and 800 °C were held out, the column-scaled condition numbers of M4 were 27.99, 10.00, and 8.92, respectively. Median RMSE values over 5000 valid stratified bootstrap replicates were 107.01, 53.96, and 115.10 µε. The corresponding median widths of the fitted-prediction bootstrap percentile intervals were 272.97, 258.76, and 685.98 µε, with a maximum width of 1226.93 µε in the 800 °C fold. These intervals indicate sensitivity to the eight-condition training sets and are not independent prediction intervals. The 76.27 µε value should therefore be interpreted as the pooled point estimate performance across the current 12 out-of-fold conditions, not as evidence of stable parameters or equivalent error in an independent heating cycle.

## 4. Discussion

### 4.1. Separation of Field Stability and Scalar Accuracy

Field smoothing and scalar accuracy diverged in both evidence streams. In the controlled simulations, isotropic scale change shifted the ROI mean with comparatively little additional spatial dispersion (Table 7 and Figure 10). In the real data, ER15 reduced spatial dispersion without reducing leave-one-temperature-out RMSE (Table 5 and Figure 7). Taken together, these results show that, under the conditions examined, strain field stability was not a sufficient surrogate for condition-level scalar accuracy in high-temperature 2D-DIC. Image stabilization produced smoother and more repeatable strain fields without a corresponding reduction in systematic scalar error, indicating that visual coherence and quantitative fidelity are distinct measurement attributes. Claims of quantitative strain accuracy should therefore be supported by independent reference measurements rather than field appearance alone. The simulations establish algorithmic responses to prescribed disturbances, not their physical origin in the experiment. This distinction is consistent with high-temperature tests in which strong DIC–extensometer correlations coexist with speckle- and temperature-dependent discrepancies [23].

### 4.2. Interpretation of Strain Radius Selection

Strain radius selection must balance noise suppression against spatial resolution [8,26,35]. The real and known-truth analyses jointly placed ER15 between the noisier ER10 setting and the more strongly smoothing ER20 setting. ER15 was therefore selected for visualization of the present uniform gauge section rather than as a universal optimum. Regions with steep gradients, including crack tips, notches, or localized necking, require renewed sensitivity analysis.

### 4.3. Interpretation of the Physics-Guided Feature

The axial–transverse feature suppresses ideal isotropic scale drift but does not correct anisotropic out-of-plane motion, lens distortion, or heat haze.

Reference targets, projected speckles, and multichannel imaging provide more direct observations of thermal errors [10,11,12,13,19,20,21,22]. Camera-noise-aware denoising with KAZE-feature-guided preregistration has likewise improved high-temperature DIC matching and field stability [18]. That approach acts at the image-registration level, whereas the present physics-guided feature operates on condition-level axial and transverse strains; the methods are complementary rather than equivalent. The common-mode feature is therefore a diagnostic and modeling variable, not a direct observer of disturbance source, and differences in optical paths and reference systems preclude numerical accuracy rankings across studies.

Real *E_xx_* also contains material response, pose effects, lens distortion, and non-common-mode errors, which explains why a direct fixed Poisson ratio correction was ineffective. Improvement occurred only when feature *f* was fitted jointly with temperature interactions and an offset. The apparent insensitivity to *ν* after refitting reflects absorption of feature rescaling by the regression coefficient and cannot identify the material’s Poisson’s ratio.

### 4.4. Internal Validation and Relevance to Harsh Environment Measurements

Condition-level leave-one-temperature-out splitting avoids leakage from random assignment of snapshots belonging to the same temperature load state. Performance was stronger for the interior 700 °C cycle than for the boundary cycles, where errors and bootstrap intervals were larger. Because temperature and cycle order are confounded, this pattern cannot be attributed uniquely to temperature transfer. The pooled estimate therefore supports within-specimen candidate model comparison rather than a transferable calibration.

Both sequences showed increased affine-removed residual RMS and reduced NCC at the high-temperature end, a trend consistent with heat haze [15,16,19,20,33,34]. However, affine removal cannot exclude lens heating, out-of-plane motion, or residual vibration. We therefore refer only to temperature-dependent non-rigid optical disturbance. Establishing causal heat haze attribution would require an in-path fixed target, synchronized flow field or temperature field measurements, and repeated heating tests.

### 4.5. Limitations and Future Validation

This study has four principal limitations. First, the quantitative results were obtained from one MA956 specimen during three separate temperature-associated cycles, without repeated cycles at the same temperature. Temperature and cycle order are therefore perfectly confounded, the four snapshots characterize within-condition technical replication only, and loaded states at a temperature share the same zero-load reference images. Second, consistent with established high-temperature 2D-DIC limitations under thermally induced out-of-plane motion [9], the monocular system cannot recover out-of-plane displacement. Only a post hoc in-plane scale estimate of 0.074205 mm/pixel was obtained; camera intrinsics, lens distortion, width tolerance, and spectral response were not quantified. Third, the MA956 batch, nominal chemical composition, heat treatment condition, specimen orientation, high-temperature paint model, and pyrometer and extensometer calibration certificate information were not completely retained; no material constitutive parameters were inferred. The retained force records showed marked post-peak relaxation, but material and system-level contributions could not be separated; strictly elastic behavior up to 2000 µε was therefore not established. The 12.6 mm ROI and 15 mm extensometer sampled different spatial supports whose exact relative placement was not recoverable. Fourth, M4 estimates four parameters from eight training conditions per fold; candidate model comparison and performance assessment use the same dataset, ROI/subset/grid choices were not evaluated systematically, and synthetic images cannot establish the real cause of heat haze. Validation after model locking using independent specimens, repeated cycles at the same temperature, and complete optical calibration is therefore required before broader application.

## 5. Conclusions

This study separated strain field visualization stability from condition-level incremental strain accuracy in high-temperature 2D-DIC. Temperature-dependent residual motion remained after affine correction, and held-out-snapshot evaluation supported affine median fusion for visualization. ER15 reduced field dispersion relative to ER10, while the known-truth analysis showed that further smoothing with ER20 reduced peak retention. ER15 is therefore a practical compromise for the present uniform gauge section rather than a transferable setting for localized strain gradients.

Controlled simulations showed that local non-affine disturbance and global scale change affect field dispersion and condition-level mean error differently. Across 20 mechanical-strain-by-magnification conditions, the common-mode feature reduced RMSE from 439.72 to 49.81 µε. In the MA956 data, the combined common-mode and temperature-conditioned model produced the lowest pooled out-of-fold RMSE of 76.27 µε, although bootstrap results indicated sensitivity to the limited training conditions. Under the tested conditions, strain field stability was therefore not a sufficient surrogate for condition-level scalar accuracy; independent reference measurements remain necessary when quantitative fidelity is claimed.

## Figures and Tables

**Table 1:** Confirmed components and settings of the experimental system.

Category	Device or Parameter	Role in this Study
Loading system	WDW-100 universal testing machine (Jilin Guanteng Automation Technology Co., Ltd., Changchun, China)	Application of uniaxial tensile load
Heating system	TH-25KVA open induction heating device (Xi’an Tianshuo Electronic Technology Co., Ltd., Xi’an, China)	Generation of the 600–800 °C environment
Camera	Photron FASTCAM NOVA S20 high-speed camera (Photron Limited, Tokyo, Japan)	Speckle image acquisition
Lens	Tokina AT-X PRO 100 mm F2.8 Macro (Kenko Tokina Co., Ltd., Tokyo, Japan)	Commercial monocular macro imaging
Optical filtering	532 nm narrow-band filter	Suppression of out-of-band thermal radiation
Illumination	White ring light (Dongguan Koma Vision Technology Co., Ltd., Dongguan, China)	Active illumination near 532 nm
Temperature reference	METIS M316 infrared pyrometer (Sensortherm GmbH, Steinbach, Germany)	Specimen surface temperature recording
Strain reference	YYHT-5 high-temperature extensometer (NCS Testing Technology Co., Ltd., Beijing, China); 15 mm gauge length	Reference strain
Image format	1024 × 1024 pixels, 8-bit BMP	Raw DIC input
Sensor	CMOS; 20.48 × 20.48 mm; 20 µm × 20 µm pixel pitch	Camera hardware record
Exposure time	0.008 s	Fixed exposure setting
Camera/interface acquisition setting	125 fps	Nominal interface setting only; the four analyzed snapshots were triggered individually without fixed intervals and were not acquired at an effective rate of 125 fps.
Working distance	Approximately 400 mm	Archived distance from the lens front to the specimen region
Filter specification	BP532-20K; center 532 ± 3 nm; FWHM 20 ± 3 nm; peak transmittance > 95%	Suppression of out-of-band radiation while retaining active illumination
Imaging settings	Lens aperture *f*/2.8; camera gain disabled	Fixed aperture and disabled gain to avoid automatic framewise grayscale changes
Estimated single-plane spatial scale	0.074205 mm/pixel (13.4761 pixel/mm)	Post hoc in-plane scale estimation from the nominal 12.5 mm specimen width in four room-temperature zero-load images
Unrecorded/uncalibrated parameters	Physical field of view; camera intrinsics, principal point, and lens distortion	Not estimated; reported as unquantified systematic error sources
Temperature trigger condition	Return to *T* ± 0.5 °C; emissivity 0.790/0.800/0.823	Manual image trigger criteria for 600/700/800 °C, respectively
Image technical replicates	Four per stable condition; manually and separately triggered; no fixed interval	Within-condition technical repeats; not a fixed-rate or hardware-synchronized image sequence
Loading process descriptor	Median absolute crosshead speed of 0.722 mm/min during the 15 s before the peak	Recovered process quantity, not a programmed machine setting

**Table 2:** Temperature–strain matrix used for quantitative analysis.

Temperature (°C)	DIC Reference State (µε)	Quantitative Strain States (µε)	Images per State	Quantitative Conditions
600	0	500, 1000, 1500, 2000	4	4
700	0	500, 1000, 1500, 2000	4	4
800	0	500, 1000, 1500, 2000	4	4

**Table 3:** Descriptive statistics of temperature-dependent non-affine residual displacement and NCC.

Sequence	*T*	Snapshot Pairs	Median residual RMS [Interquartile Range (IQR)] (pixel)	Endpoint Change	Median NCC [Interquartile Range (IQR)]
Freely suspended	25 °C	6	0.02805 [0.02689, 0.02942]	–	0.99139 [0.99133, 0.99141]
Freely suspended	600 °C	6	0.02805 [0.02739, 0.02910]	–	0.99083 [0.99064, 0.99105]
Freely suspended	700 °C	6	0.03581 [0.03543, 0.03673]	–	0.98947 [0.98943, 0.98956]
Freely suspended	798 °C	6	0.04007 [0.03921, 0.04189]	+42.9%	0.98653 [0.98646, 0.98661]
Zero load	600 °C	6	0.03109 [0.02858, 0.03127]	–	0.98972 [0.98897, 0.99054]
Zero load	700 °C	6	0.03375 [0.03186, 0.03422]	–	0.98960 [0.98889, 0.98997]
Zero load	800 °C	6	0.04152 [0.04107, 0.04206]	+33.5%	0.98663 [0.98623, 0.98692]

**Table 4:** Results of the paired held-out-snapshot technical evaluation. Each temperature contains 20 evaluations, for a total of 60. Percentages are median changes relative to a raw single snapshot; the final two columns report the number of improvements within each temperature.

Method	Temperature	RMS Change	NCC Change	RMS Improvements (20 per Temperature)	NCC Improvements (20 per Temperature)
Affine three-snapshot template	600 °C	14.00% reduction	+0.00265	19/20	20/20
Affine three-snapshot template	700 °C	10.03% reduction	+0.00277	18/20	20/20
Affine three-snapshot template	800 °C	11.39% reduction	+0.00877	20/20	20/20
Non-affine-corrected single snapshot	600 °C	8.76% reduction	+0.00011	18/20	20/20
Non-affine-corrected single snapshot	700 °C	6.74% reduction	+0.00005	19/20	19/20
Non-affine-corrected single snapshot	800 °C	8.55% reduction	+0.00065	19/20	20/20
Non-affine three-snapshot fusion	600 °C	2.18% increase	+0.00267	11/20	20/20
Non-affine three-snapshot fusion	700 °C	1.38% increase	+0.00284	8/20	20/20
Non-affine three-snapshot fusion	800 °C	0.68% reduction	+0.00874	12/20	20/20

**Table 5:** ER10/ER15 sensitivity in the real MA956 data.

Metric	ER10 (µε)	ER15 (µε)	Relative Change with ER15	Interpretation
Spatial SD of loaded fields	554.67	428.80	−22.7%	Reduced field dispersion
*P*_95_ minus median of loaded fields	814.27	678.90	−16.6%	Narrower upper tail
Same-load *P*_95_ minus median	254.03	138.31	−45.6%	Reduced local high-value tail
SD of four frame means	94.11	93.91	−0.2%	Scalar repeatability nearly unchanged
Leave-one-temperature-out RMSE	75.53	76.27	+1.0%	Temperature-held-out RMSE nearly unchanged

**Table 6:** Known-truth evaluation of ER10, ER15, and ER20 on controlled synthetic strain fields.

Setting	ER	Synthetic Fields	Field RMSE (µε)	*P*_95_ Absolute Error (µε)	Gradient Field Correlation	Localized Field Correlation	Localized *P*_95_ Peak Retention
SR20_ER10	10	9	290.3	547.8	0.916	0.827	85.0%
SR20_ER15	15	9	220.1	423.8	0.960	0.874	76.6%
SR20_ER20	20	9	188.0	385.2	0.978	0.881	68.2%

**Table 7:** Four-frame fusion results for the field stability/scalar error decoupling simulation; the mechanical axial strain ground truth was 1000 µε in every condition.

Condition	Local Non-Affine Disturbance	Scale Change	Fused Mean (µε)	Change from Clean Condition (µε)	Spatial SD (µε)	Scalar Bias (µε)
Clean	No	0 ppm	946.61	0.00	134.73	−53.39
Local disturbance only	Yes	0 ppm	944.03	−2.58	141.33	−55.97
Scale change only	No	300 ppm	1330.32	+383.71	141.39	+330.32
Combined disturbances	Yes	300 ppm	1310.64	+364.03	148.82	+310.64

**Table 8:** Quantitative summary of magnification-induced apparent strain and common-mode-feature simulations.

Simulation Evidence	Metric	Value	Interpretation
11 pure-scale conditions	Theory measurement slope	0.9805	Near-unit response
11 pure-scale conditions	Intercept	0.57 µε	Small fixed offset
11 pure-scale conditions	*R* ^2^	0.9987	High linear agreement
11 pure-scale conditions	Fit RMSE	35.35 µε	Finite algorithmic error remains
20 mechanical-scale conditions	Axial RMSE	439.72→49.81 µε	88.67% reduction
20 mechanical-scale conditions	Mean across-scale span at fixed load	1192.34→2.64 µε	Ideal common-mode term suppressed
Poisson ratio sensitivity	RMSE for *ν* = 0.25–0.35	48.00–85.90 µε	Result depends on assumed parameter

**Table 9:** Leave-one-temperature-out internal assessment performance of the temperature-conditioned candidate model.

Held-Out Temperature	Assessment Type	Bias (µε)	MAE (µε)	RMSE (µε)	Maximum Absolute Error (µε)	*R*^2^ (Pooled only)
600 °C	Boundary holdout	48.79	90.76	104.14	153.12	—
700 °C	Within-range interpolation	−28.48	28.48	36.73	61.82	—
800 °C	Boundary holdout	67.90	67.90	72.51	99.42	—
All held-out predictions	Pooled	29.40	62.38	76.27	153.12	0.9815

## Data Availability

The raw experimental data and condition-level data supporting the reported ER10 and ER15 results are provided in the Supplementary Materials, together with essential processing and controlled simulation parameters. The analysis software is being prepared for intellectual-property registration and is therefore not publicly distributed at this stage. Requests for access to the analysis software and additional intermediate files will be considered by the corresponding author, subject to institutional approval and applicable intellectual-property restrictions.

## Supplementary Materials

The following supporting information can be downloaded at: https://www.mdpi.com/article/10.3390/s26196333/s1, Supplementary Data S1. Raw experimental images and corresponding extensometer and temperature records; Supplementary Data S2. Image condition index, condition-level DIC inputs, extensometer references, ER10/ER15 out-of-fold predictions, residuals, and summary metrics; Supplementary File S1. README and essential processing and controlled-simulation parameter files. The Ncorr, ECC registration, and Farnebäck optical-flow methods associated with these files are described in Section 2.3, Section 2.4 and Section 2.5 and cited in References [25,31,32].
